# How to improve worldwide early enteral nutrition performance in intensive care units?

**DOI:** 10.1186/s13054-018-2188-5

**Published:** 2018-11-21

**Authors:** Arthur Raymond Hubert van Zanten

**Affiliations:** Department of Intensive Care Medicine, Gelderse Vallei Hospital, Willy Brandtlaan 10, 6716 RP Ede, The Netherlands

Early enteral nutrition (EEN), typically started within 48 h after ICU admission, is recommended to be superior over delayed enteral nutrition and parenteral nutrition. The ESICM Working Group on Gastrointestinal Function provided clinical practice guidelines on EEN and suggested to initiate it at a low rate, as beneficial effects regarding infection prevention have been demonstrated in critically ill patients, as well as in patients with severe acute pancreatitis and after gastrointestinal (GI) surgery [1].

Delaying EN was only suggested in patients with uncontrolled shock (when hemodynamic and tissue perfusion goals are not met despite fluids and vasopressors), in uncontrolled hypoxemia and acidosis, uncontrolled GI bleeding, overt bowel ischemia (occlusive or non-occlusive), bowel obstruction (mechanical ileus), abdominal compartment syndrome, gastric aspirate volume (GRV) > 500 ml/6 h or high-output fistula if reliable distal feeding access is not achievable. These temporary contraindications are not present in the majority of ICU patients. The recommendations have been adopted in the new nutrition guidelines for adult ICU patients by the European Society for Clinical Nutrition and Metabolism (ESPEN) as of September 2018, soon to be published in *Clinical Nutrition* (P. Singer, Madrid, personal communication ESPEN Congress].

## What is the actual worldwide early enteral feeding performance?

In a cross-sectional, observational study in eight Latin-American countries among 1053 patients from 116 hospitals, caloric intake failed to meet the daily target in 40% of patients on day 1 [2]. Other observations were reported from the Nutrition Day ICU study concerning a 7-year worldwide prevalence study of nutrition practices in ICUs; more than 40% of the patients were not fed during the first day [3].

## What is the actual early enteral feeding performance in China?

In a recent study published in *Critical Care*, an even more significant gap between the EEN recommendations and the actual feeding performance in Chinese ICUs was reported [4, 5]. Only one-third of the patients received EN within two days. The proportion of subjects receiving > 80% of the energy target was only 4.8% after three days and 8.2% after one week. Significantly lower EN intakes among male patients were observed, possibly due to their higher body weight. However, when the median volume of EN administered of precisely 1000 ml in all patients is combined with the findings of the gender effect, it seems that a standard dose of 1000 mL is provided to many patients, irrespective of their estimated caloric or protein needs. This median volume probably reflects a more or less one size fits all strategy. Similar observations were done in other countries [3]. These findings suggest that no personalized strategies are implemented and the guidelines are not followed.

## How to improve worldwide performance?

In a review by Kozeniecki and coworkers [3], many barriers and solutions to delivery of ICU nutrition therapy are addressed. Based on national or local opinions, however, protocols and implementation strategies have to be adjusted to overcome the challenges and improve performance. Let’s take China as an example.

## How to overcome the barriers in China?

Based on a survey among 162 physicians from 45 ICUs in China we know that the attitude towards American guidelines is positive; 94% of the respondents consider nutrition therapy very important and 80% mention using the American guidelines [6]. However, more negative opinions were noticed for supplemental parenteral nutrition (59%) and the cut-off values for GRV (41%). These negative opinions may reflect the preference of Chinese physicians to start with early parenteral nutrition and later advance to EN.

When we consider the average weight of Americans and Europeans to be 81 and 71 kg, respectively, and compare these with the weight of the Chinese patients (65 kg) in the Juan Xing study, this could imply that the cut-off for GRV in China should be 10–20% lower, thus 400–450 mL/6 h [4, 7]. In this study, however, the median GRV was 0 mL (IQR 0–50 mL) and therefore does not indicate a threshold problem.

The configuration of the EN devices and staffing is adequate in China’s tertiary hospitals treating neurocritical care patients [8]. Of the ten guidelines for EN practices, setting the energy target, choosing the EN tube, and monitoring the patient showed poor compliance, 56.2%, 30.0%, and 38.9%, respectively.

## Create change

**Fig. 1:**
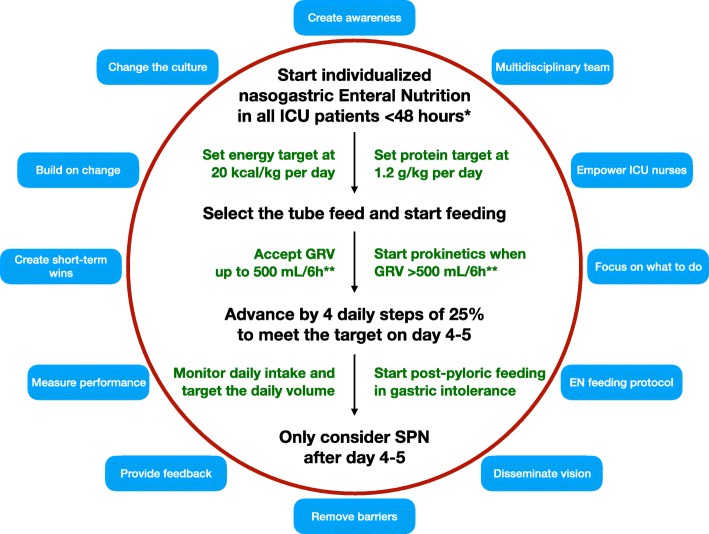
Strategies to improve early enteral feeding performance. *When oral feeding is impossible or supposed not to achieve the targets. Delay enteral nutrition only in patients with uncontrolled shock (when hemodynamic and tissue perfusion goals are not met despite fluids and vasopressors), in uncontrolled hypoxemia and acidosis, uncontrolled GI bleeding, overt bowel ischemia (occlusive or non-occlusive), bowel obstruction (mechanical ileus), abdominal compartment syndrome, gastric aspirate volume (*GRV*) > 500 ml/6 h or high-output fistula if reliable distal feeding access is not achievable. **Modified cut-off for GRV for the Chinese population: 400 ml/6 h. Abbreviations: *GRV* gastric aspiration volume, *EN* enteral nutrition, *GI* gastrointestinal, *SPN* supplemental parenteral nutrition

Based on the rules of change management the first step is to create the awareness that the performance is not in line with international recommendations. The results from the international and Chinese observational studies suggest that many ICU patients do not benefit from EEN. In Fig. 1 a simple strategy to improve EEN performance is suggested. A slightly lower GRV is suggested for the Chinese population; however, all the other improvement steps could be applied throughout the world.

## Is there evidence that enteral nutrition improvement strategies work in China?

Nasogastric tube feeding of critically ill patients in a Chinese neurosurgical ICU was markedly improved after the implementation of a best practice strategy [9]. Collaboration, education, monitoring, and a reward system were the essential elements driving this success.

Also, EN protocols may help. The effectiveness of an EN protocol was studied in a before and after study among Chinese critically ill patients. The proportion of enteral feeding improved significantly after protocol implementation [10].

Furthermore, improved outcomes in China have been suggested with EEN. In a propensity-matched study, EEN therapy in patients with emergency intestinal surgery significantly reduced the total number of patients with complications, early ileus, time to first defecation, length of hospital stay, mortality, and increased 28-day ICU-free days [11].

## Conclusion

A multifaceted approach is warranted to improve the EEN performance in ICUs all over the world and in particular in China, involving all relevant stakeholders such as medical doctors, nutritionists and dieticians, nurses, healthcare assistants and, last but not least, patients and their families. This paper is an appeal to the international critical care, nutrition, and administrative leadership to initiate the change and improve enteral feeding practice in ICUs. Therefore, let’s cooperate and follow the wisdom of the ancient Chinese proverb: “Talk does not cook rice”.
